# A comparative proteomic study of sera in paediatric systemic lupus erythematosus patients and in healthy controls using MALDI-TOF-TOF and LC MS–A pilot study

**DOI:** 10.1186/1546-0096-10-24

**Published:** 2012-08-17

**Authors:** Anita Rana, Ranjana W Minz, Ritu Aggarwal, Sadhna Sharma, Neelam Pasricha, Shashi Anand, Surjit Singh

**Affiliations:** 1 Department of Immunopathology, Post Graduate Institute of Medical Education and Research (PGIMER), Chandigarh, 160012, India; 2 Department of Biochemistry, Post Graduate Institute of Medical Education and Research (PGIMER), Chandigarh, 160012, India; 3 Paediatric Allergy Immunology Unit Advanced Paediatrics Centre, Post Graduate Institute of Medical Education and Research (PGIMER), Chandigarh, 160012, India

**Keywords:** Paediatric systemic lupus erythematosus, Two dimensional poly acryl amide gel electrophoresis, Matrix assisted laser desorption ionization time of flight, Liquid chromatography mass spectrometry, Retinol binding protein, Leiomodin 2, Transthyretin, Apolipoprotein A1

## Abstract

**Background:**

Paediatric systemic lupus erythematosus (pSLE) exhibits an aggressive clinical phenotype with severe complications and overall poor prognosis. The aim of this study was to analyse differential expression of low molecular weight (LMW) serum protein molecules of pSLE patients with active disease in comparison to sera of healthy age matched controls. Further, some of the differential expressed spots were characterised and identified by Matrix Assisted Laser Desorption Ionization Time of Flight Mass Spectrometry (MALDI-TOF-MS) and liquid chromatography (LC-MS).

**Methods:**

2D-PAGE was performed using pooled sera of active pSLE and age matched healthy controls. Gels were silver-stained and differentially expressed protein spots were detected by automated image master platinum 2D software. 79 ± 17 protein spots were detected for control gels and 78 ± 17 protein spots for patient gels. Of these eleven protein spots were selected randomly and characterized by MALDI-TOF MS (five protein spots) and LC MS (six protein spots) techniques.

**Results:**

Out of the 11 protein spots, 5 protein spots were significantly upregulated viz., leiomodin 2 (LMOD2); epidermal cytokeratin 2; immunoglobulin kappa light chain variable region; keratin 1 and transthyretin (TTR). Three protein spots were significantly down regulated e.g.**,** apolipoprotein A1 (APOA1); chain B human complement component C3c; campath antibody antigen complex. Two protein spots (complement component C3; retinol binding protein (RBP) were found to be expressed only in disease and one protein spot cyclohydrolase 2 was only expressed in controls.

**Conclusions:**

We conclude that 2-D maps of patients with active pSLE and controls differ significantly. In this pilot study, using proteomic approach we have identified differential expressed proteins (of LMW) e.g., RBP, LMOD 2, TTR, Component C3c Chain B and APO A1. However, in future, further studies need to confirm the physiological and pathological role of these proteins in similar cohorts of pSLE.

## Background

Systemic lupus erythematosus (SLE) is chronic autoimmune disease with unknown aetiology and complex pathogenesis. pSLE is more severe and exhibits a more aggressive clinical course than adults. Genuine difficulties are faced in diagnosing, classifying, and treating pSLE.

A proteomic approach is necessary to find proteins expressed in the disease states, which may serve as diagnostic or prognostic marker, or serve as targets for therapeutics. Such an approach has yielded newer diagnostic proteins in the other autoimmune diseases viz., myelin protein Po in autoimmune inner ear disease [1], heterogeneous nuclear ribonucleoprotein A2/B1 in autoimmune hepatitis, α-enolase in Behcet’s disease [2]**,** α-enolase and its citrullinated molecule in rheumatoid arthritis [3-7]. Traditional methods, such as ELISA or western blotting, can only study several known proteins simultaneously. On the contrary, 2-dimensional poly acryl amide gel electrophoresis (2D-PAGE) combined with protein identification by mass spectrometry (MS) which can help to identify a very large number of unknown proteins [8,9]. Proteomic approaches in autoimmune diseases are also required in addition to gene expression studies as the latter has many limitations. First, diseases manifest not at the level of RNA transcription, but rather at the level of the protein. Second, there is a frequently non predictive correlation between RNA expression and protein expression and function [10,11]. Messenger RNA undergoes a variety of processing events that can profoundly affect cell phenotype yet are not revealed in current transcriptional profiles [12-14]. Third, protein function can be regulated by posttranslational modifications by enzymes such as kinases or proteases.

In the present study, we attempted a proteomic approach and performed comparative protein analysis of sera of pSLE and healthy control subjects by 2D-PAGE followed by Matrix Assisted Laser Desorption Ionization and time of flight (MALDI-TOF) and Liquid chromatography-mass spectrometry (LC-MS) for the identification and characterization of limited number of some significantly expressed proteins spots. The function and classification of these proteins were then determined by searching the public database.

## Methods

Forty consecutive pSLE patients (5–16 years of age) were recruited from the Division of Allergy Immunology, Advanced Paediatrics Centre, Post Graduate Institute of Medical Education and Research (PGIMER), Chandigarh, India. The diagnosis of pSLE patients was based on the American College of Rheumatology (ACR) criteria for classification [15]. Twenty healthy and age-matched volunteers served as controls. Study was carried out between March 2007 and April 2011. The mean age of the pSLE patients was 15.2 years (range 5–16). The median SLE Disease Activity Index (SLEDAI) scores were 15 (range of period 4–26**)**[16]**.** All patients were positive at a significant titer for antinuclear antibodies included in the study. Patients with overlap syndrome were excluded from the study. All patients were on treatment using standard protocols incorporating, glucocorticoids and immunosuppressive agents (e.g. cyclophosphamide and azathioprine). Patient information in regard to demographic data, cumulative clinical features, serological profile, and medications were retrieved from medical records (shown in Table 1). The study protocol was approved by the Institute Ethics Committee. All parents/guardians of pSLE patients gave a written informed consent. 

**Table 1:** Demographics and clinical parameters of paediatric lupus patients and control groups

**Variables**	**pSLE (N = 40)**^**1**^	**Controls (N = 20)**^**1**^	**p value**^**2**^
**Age (years**)			
Median	12	13	0.213
Range	5-16	10-15	
Sex (Female/male)	34/6	15/5	0.326
**Disease duration (months)**			
Median	8	NA	
Range	1-96	NA	
**SLEDAI score**			
Median	15	NA	
Range	4-26		
**Clinical manifestations**^**1**^			
Cutaneous	25 (62.5)	NA	
Skin Biopsy	13 (32.5)	NA	
Renal	24 (60)	NA	
Renal Biopsy	10 (25)	NA	
Haematological	18 (45)	NA	
Oral ulcers	12 (30)	NA	
Musculoskeletal	12 (30)	NA	
Neurological	10 (25)	NA	
**Medications used**^**1**^			
I. Prednisolone	11(27.5)		
II. Prednisolone and anti-malarials (chloroquine and hydroxychloroquine)	19 (47.5)		
III. Immunosuppressive therapy ^3^	10 (25)		

Abbreviations: *SLEDAI* SLE disease activity index; Lupus nephritis class based on international society of nephrology/renal pathology society classification criteria (*ISN/RPS*), *NA* not applicable, ^1^ Values are represented as either median & range or Number (percentage): N (%); ^2^p ≤ 0.05 as significant; ^3^(methotrexate, azathioprine, mycophenolate mofetil, cyclosporine and cyclophosphamide).

### Serum separation and protein extraction

Sera were separated from whole blood of pSLE and healthy volunteer children. Blood samples were collected (in autoclaved glass tubes) and left for 1 h at 37°C to allow it to clot. Further, samples were left at 4°C overnight to allow the clot to contract. Using a glass pasteur pipette, the clot was loosened carefully from the side of the tube. A care has been taken to not to lyse the red cells as they cannot then be separated from the serum. Serum was centrifuged at 4000 rpm for 20 min at 4°C. Serum was removed from the clot by gently pipetting off into a clean tube. It was stored at −20°C.

### Comparative protein analysis of sera of pSLE and healthy control subjects by 2-Dimensional Poly acryl amide gel electrophoresis (2D-PAGE)

Protein concentration from sera was estimated by BIORAD protein assay (modified Bradford assay). Mean concentration of protein in sera from control sample was estimated as 66.9 mg/ml and from SLE disease sample as 65 mg/ml. Proteins (100 μg) were cleaned up using 2-D Clean-Up Kit (GE Healthcare) also as described by the manufacturer. The dried protein pellet was then redissolved in 250 μl swelling solution containing 7 M urea, 2 M thiourea, 2% CHAPS, 20 mM DTT and 0.5% IGP buffer pH 3–10. 2-D electrophoresis Immobilized pH gradients (IPG) isoelectric focusing (IEF) of the protein (100 μg) was performed according to the guideline of IPG phor IEF system (GE Healthcare, Piscataway, NJ, USA). Thirteen centimetre immobilized pH 4–7 gradient IPG strips (GE Healthcare, Piscataway, NJ, USA) were chosen, and IEF were run at 20°C using the following conditions: 12 h rehydration, 1 h at 500 V, 1 h at 1000 V and at 8000 V to a total voltage × time of 60 KVh. Strips were then equilibrated by incubating in 6 M urea, 30% glycerol, 2% sodium dodecyl sulfate (SDS), 50 mM Tris/Cl pH 8.8 (SDS equilibration buffer solution) containing 1% DTT, 10 ml per strip for 15 min at room temperature, followed by an incubation for 15 min at room temperature in 10 ml per strip SDS equilibration buffer solution containing 2.5% iodoacetamide and a trace of bromophenol blue. Strips were then placed on top of 12.5% SDS polyacrylamide gel electrophoresis gels and electrophoresed at 30 milli ampere per gel until the bromophenol blue dye front had reached the bottom of the gel. Each sample was run on duplicate gels.

### Silver staining and image analysis

Gels were silver stained using the improved protocol compatible with mass spectrum **analysis**[17]**.** After gel scanning, images were analyzed using Image Master 2D Platinum 5.0 software (GE Healthcare, Piscataway, NJ, USA). For each gel, the preliminary analysis included protein spot detection, editing, filtration and quantification. Spots were quantitated as a fraction of the total volume of protein spots on the gel. One typical control gel with the most protein spots was then set as reference gel, to which all the other gels were matched. Match rates of each group of gels were calculated automatically by the software. Class report analysis was run on the software and each spot group with a between-class ratio of more than two or less than 0.5 was statistically analyzed using the Mann–Whitney rank sum test, two-tailed.

### MALDI-TOF-TOF-MS and database searching

The differentially expressed protein spots were excised and collected followed by washing in ex-ion water and destaining through acid ammonium carbonate of 50 mmol/L plus 50% acetonitrile. Samples were then treated with trypsin overnight at 37°C. The peptide segments were drawn through 0.1% trifluoroacetic acid (TFA) plus 50% acetonitrile and dried by nitrogen gas. The loading samples were covered by matrix solution (0.1% TFA and 50% acetonitrile) of 0.8 L. After air-drying, the samples were analyzed by peptide mapping fingerprint (PMF) and MALDI-TOF-TOF-MS, which was rectified by the inner markers including the base peak and the inscribed trypsin peak. The obtained peptide mass fingerprints were used to search through the SWISS-PROT and NCBInr database by the Mascot search engine, with which MS/MS ion search was also finished. In addition, the amino acid sequences of the peptides were deduced with the peptide sequencing program MasSeq.

## Results

### 2-D electrophoresis maps of sera in control and patients with pSLE

**Figure 1:**
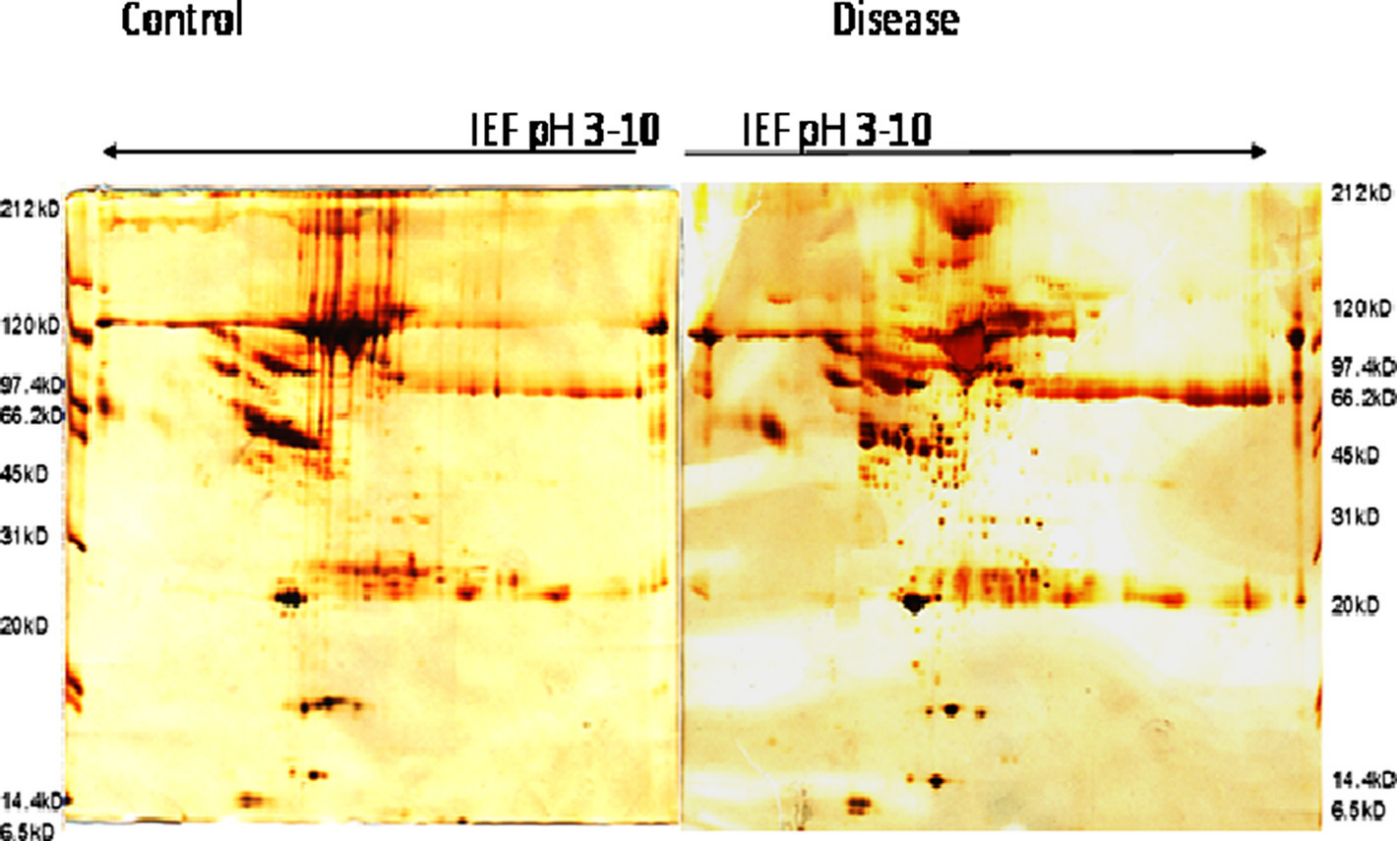
**Two dmensional PAGE gel picture of SLE control and patient usinf serum, showing differentially espressed spots and selected differential spots were further MALDI and LC-MS analysis (IEF at pH 3-10, IPG strip-17cm).** The molecular weight of the protein markers are indicated on the sides of each gel.

Using gel image analysis software Image Master 2D Platinum 5.0, we compared 2D maps of sera between patients with SLE and healthy controls. The images were found to be similar either between gels or between groups. There were approximately more than 500 spots that could be visualized manually in the 2D gel images of both pSLE patients and controls. After comparing with the reference gel, match rates of 63% ± 4% and 62% ± 4% were for control and patient gels, respectively. Simultaneously, 79 ± 17 spots were detected for control gels and 78 ± 17 for patient gels. Figure 1 shows a typical control gel and a typical patient gel, respectively.

### Differentially expressed proteins between patients with SLE and controls

**Figure 2:**
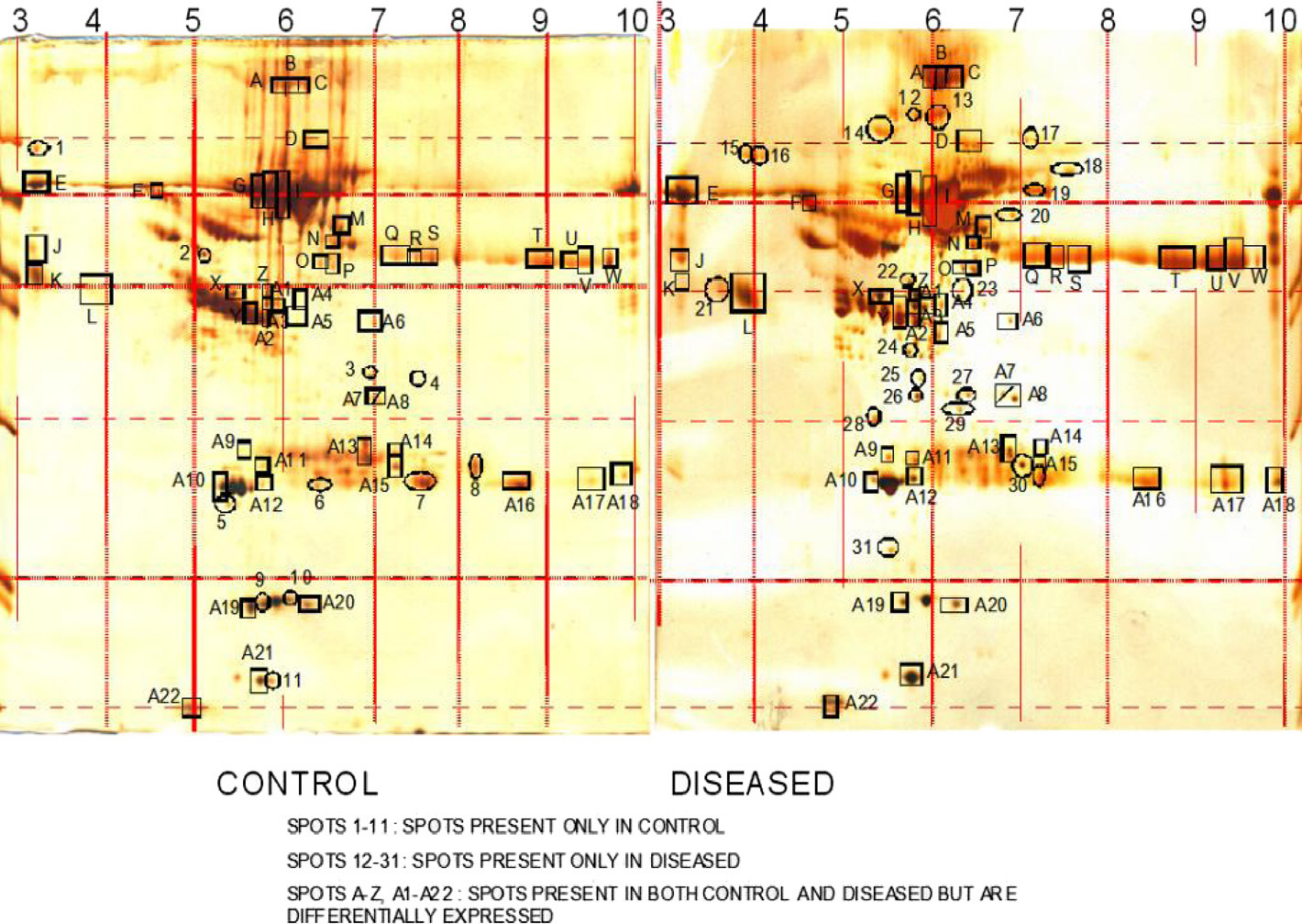
Two dimensional poly acryl amide gel pictures of control and disease using sera shows spots categorization-Spots 1-11: spots present only in control, spots 12-31: Spots present only in diseased, spots A-Z, A1-A22: Spots present in both control and diseased but are differentially expressed.

**Figure 3:**
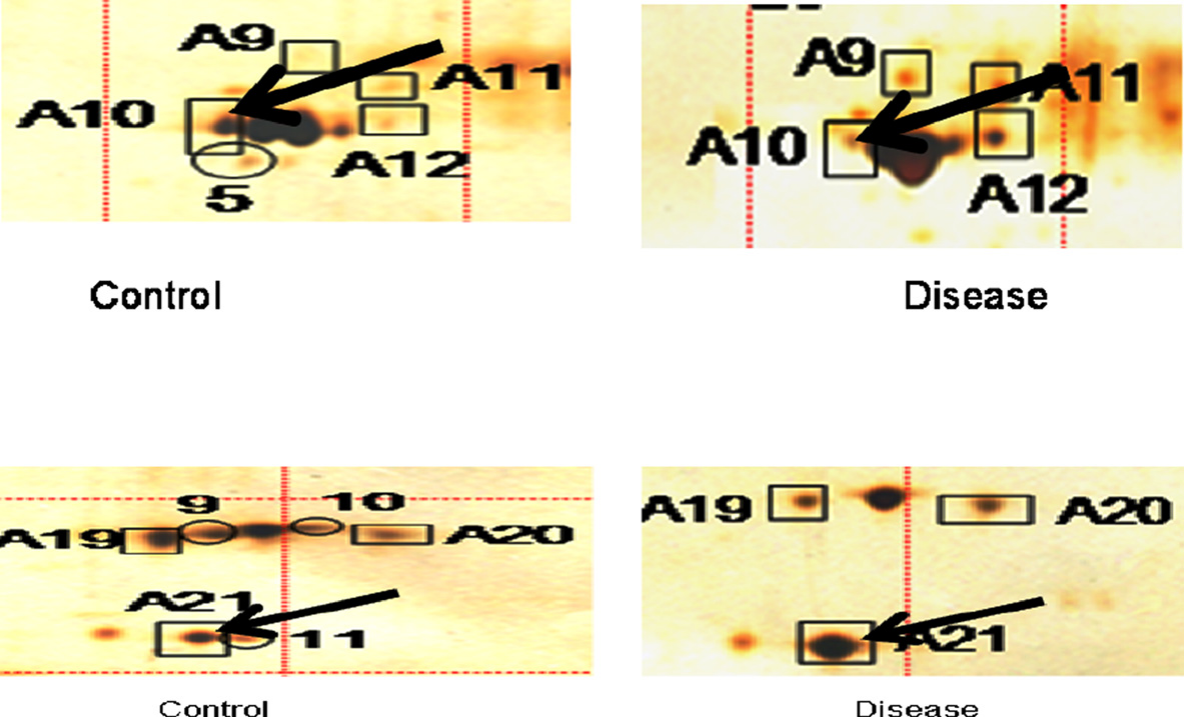
**A Enlarged areas of the control and patient gels showing a comparison of the expressions of spot A10 Apolipoprotein A1 (down regulated in disease as indicated by black arrow) in these gels.** On the left is a control gel and on the right is a patient gel. **B** Enlarged areas of the control and patient gels showing a comparison of the expressions of spot A21 Transthyretin Variant del 122 was over expressed in disease gel (indicated by black arrow) in these gels. On the left is a control gel and on the right is a patient gel.

To identify proteins that were differentially expressed in one group with respect to the other, we compared the value of expression of each spot in the patient group as a ratio of the value of expression of that spot in the control group using the ‘class report’ command of Image Master 2D Platinum 5.0 for each spot groups. This resulted in 79 spot groups with a between-class ratio of more than one or less than 0.5. The average value for these spots on the two duplicate gels per individual was calculated, and these average values were then compared using the Mann–Whitney rank sum test, two-tailed. Out of 79 spots, we selected 11 spots for the identification and characterization by MALDI-TOF and LCMS analysis. These protein spots were chosen randomly from the low molecular weight region, preferably at a molecular mass lower than 20 KD, as can be seen from their location and labeling in the patent and control gels (Please see Figure 1). LMW proteome, low abundant proteins are especially important for biomarker discovery in SLE. Because this region contains biologic mediators (cytokines, chemokines, growth factors) that are expected to be involved in disease pathogenesis. Some of these mediators have been found in active SLE renal tissue and may reflect organ-specific tissue injury [18]**.** 2D PAGE followed by MS has been shown earlier to detect urine proteins that discriminate between ISN/RPS lupus nephritis classes [19]. In our cohort, all these 11 spots had p value of less than 0.05. There were spots which were showing low score in MALDI-TOF. For them we have to proceed further for LC-MS/MS analysis. Six protein spots were up-regulated (labelled as A −11, A-16, A-17, A-18, A-21), and five protein spots were down-regulated (labelled as A-10, A-13 and W) in patients with pSLE (Figure 2). D19 and D31 were the spots highly expressed in the patients. The median volume of the eleven spots in the two groups was also calculated. Figures 3A and 3B are enlarged areas of the control and patient gels showing a comparison of the expressions of spot A10 and A21 in these gels respectively. 

### Identification and characterization of proteins by Mass Spectrometry (MALDI-TOF- MS and LCMS)

**Figure 4:**
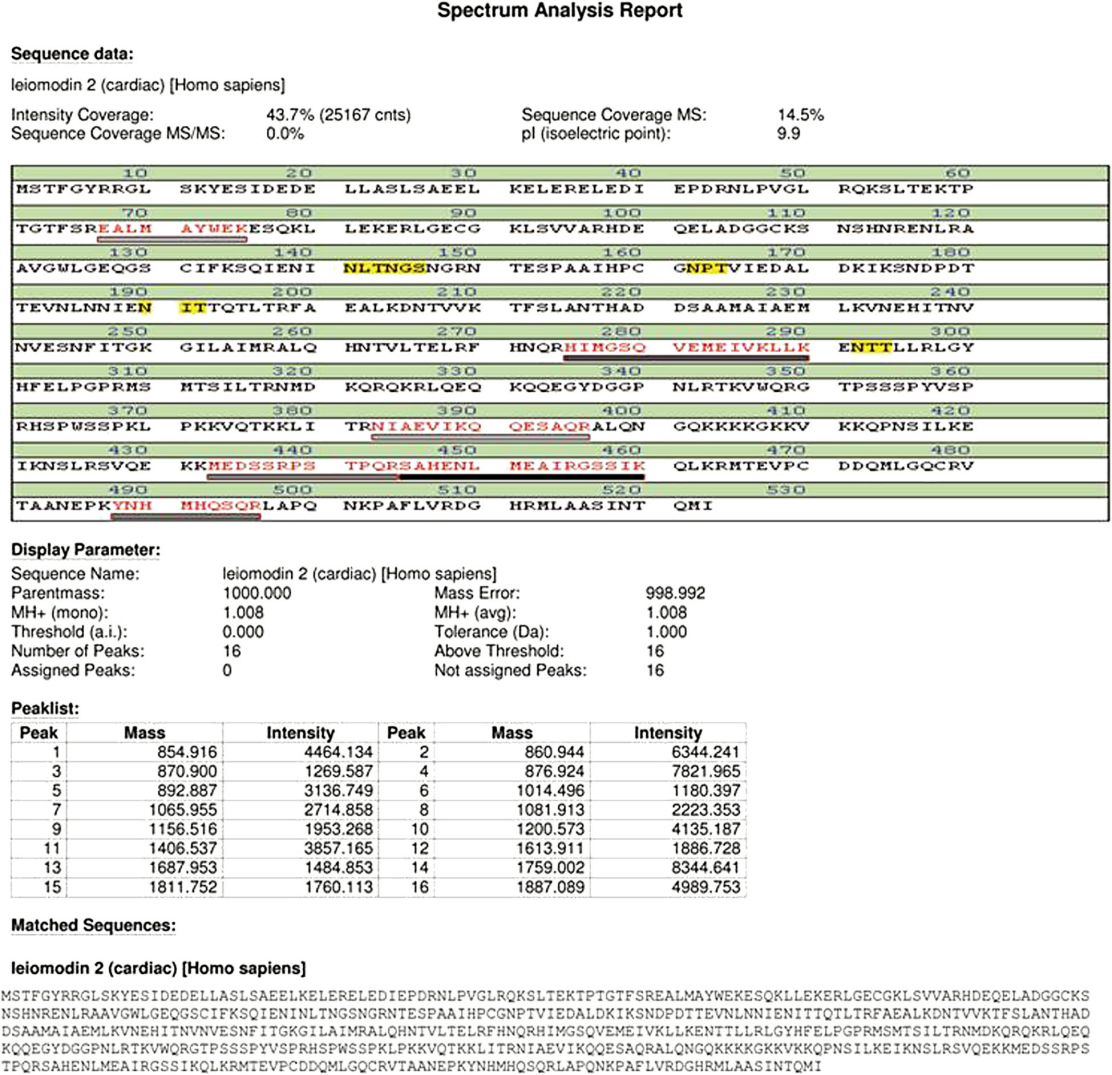
MALDI MS analysis report for a representative protein spot.

Differentially expressed protein spots were excised from the gels, digested with trypsin and the proteolytic fragments analyzed by MALDI-TOF mass spectrometry as described in materials and methods section. We identified eleven proteins, which are summarized in Table 2 &3. Differentially expressed protein spots, their fold regulation values, spot categorization (U, up-regulated in pSLE; D, down-regulated in pSLE), molecular weight (MW) in daltons (Da), isoelectric point (pI), sequence coverage (%) of matching peptides and their spectrum analysis are shown in Tables 1 and 2 and Figure 4.

**Table 2:** Spectrum analysis (MALDI-TOF & LC-MS) results 11 spots

**S. No**	**Spot ID**	**Accession no (Database = NCBI nr)**	**Protein sequence name**	**Molecular mass (Dalton)**	**pI (Isoelectric point)**	**Sequence coverage MS**	**Intensity coverage**	**Score**	**No of peaks**
1.	A-10 (LC-MS)	gi|178775	Pro-apo-lipoprotein [Homo sapiens]	28944	5.13	7%		213	
2.	A-11 (MALDI-TOF)	gi|51095091	Leiomodin 2(Cardiac) [Homo sapiens]	59366	9.9	14.5%	43.7% (25167 cnts)	69.4	16
3.	A-13 (MALDI-TOF)	gi|78101270	Chain B, Human Complement Component C3C [Homo sapiens]	21596	5.8	18.1%	36.3% (13319 cnts)	68.0	17
4.	A-16 (LC-MS)	gi|269849769	Epidermal cytokeratin 2 [Homo sapiens]	59020	5.13	7%	-	171	-
5.	A-17 (LC-MS)	gi|29836906	Immunoglobulin kappa light chain variable region [Homo sapiens]	8811	8.65	20%	-	62	-
6.	A-18 (LC-MS)	gi|7331218	Keratin 1[Homo sapiens]	66149	8.16	3%	-	63	-
7.	A-21 (MALDI-TOF)	gi|212374952	Chain A, Crystal structure of Transthyretin Variant del 122[Homo sapiens]	13798	5.35	48.8%	33.4% (18627 cnts)	85.4	15
8.	W (LC-MS)	gi|5542161	Chain H, 1.9a Structure Of The Therapeutic Antibody Campath-1 h Fab In Complex With A Synthetic Peptide Antigen[Homo sapiens]	23740	9.03	6%	-	105	
9.	C7 (MALDI-TOF)	gi|222418558	Bi-functional Methylene tetra hydrofolate de-hydrogenase/cyclohydrolase 2[Homo sapiens]	37463	10.0	21.3%	37.3% (18238 cnts)	67.00	14
10	D19 (MALDI-TOF)	gi|119589476	Complement component C3, partial [Homo sapiens]	144417	7.8	7.5%	51.0% (34497 cnts)	67.2	21
11.	D31 (LC-MS)	gi|18088326	Retinol binding protein[Homo sapiens]	23371	5.77	10%	-	112	-

**Table 3:** Summary of differentially expressed protein spots showing Fold-regulation (Ratio: Diseased/Control as calculated by PD Quest Software)

**S. No**	**Spot-No.**	**Control-Intensity**	**Diseased-Intensity**	**Fold regulation**	**P value**
1	A-10	1516.72	873.84	0.58	0.011
2	A-11	411.61	597.71	1.45	0.021
3	A-13	2129.56	1635.24	0.77	0.012
4	A-16	2812.67	3452.11	1.23	0.001
5	A-17	1720.75	2687.49	1.56	0.002
6	A-18	1074.08	1666.51	1.55	0.004
7	A-21	1528.01	2347.67	1.54	0.006
8	W	1731.3	1082.16	0.63	0.019
9	Control-7	3734.48	0	0.00	-
10	Disease-19	0	2448.43	0.00	-
11	Disease-31	0	780.03	0.00	-

## Discussion

Recently, the proteomic approach has been successively used to identify the biomarkers in skin, kidney or plasma of SLE patients [5,6,20,21]. However, to the best of our knowledge, no such study has been reported on pSLE patients using sera from north India. In this preliminary study, we attempted a proteomic approach such as 2-DE and MALDI-TOF MS and LC-MS to find candidate molecules in sera of pSLE patients with active disease in comparison to age matched healthy controls.

On evaluation of our data five differentially expressed protein spots (within low molecular weight region) were identified: Retinol-binding protein, Leiomodin2, Transthyretin, Component C3c Chain B and Apolipoprotein A1 (Table 2).

Retinol binding protein (RBP), a carrier protein that binds to retinol, was found to be over expressed in sera of pSLE patients but not in controls. Its role has been previously described to be associated with nephritis especially in children [22,23]. It has also been described as a urinary marker of proximal renal tubular dysfunction [24-26]. We have described the increased expression of RBP protein spots in pooled sera of pSLE patients with active disease and compared them with controls. It would be important to quantitate this protein and discriminate between different sub-groups of patients with pSLE, for example active and non-active disease and with other clinical manifestations (e.g. Lupus nephritis class based on international society of nephrology/renal pathology society classification criteria (WHO/ISN/RPS) which was not covered in this study.

Leiomodin 2, also known as cardiac (C) - Lmod2 or Lmod 2, was overexpressed in sera of pSLE patients. It is a 547 amino acid protein that is specifically expressed in heart and skeletal muscles. Leiomodin 2 is encoded by a gene that is located near the hypertrophic cardiomyopathy locus CMH6 on chromosome 7, suggesting that Lmod2 may be involved in that disease process. Tsukada et al. have identified a function of Lmod2 in the regulation of thin filament lengths. Over expression of Lmod 2 results in loss of Tmod1 assembly and elongation of the thin filaments from their pointed ends. Primary function of Lmod2 is to maintain thin filament lengths in the mature heart. It has been shown to be essential for efficient contractile activity of heart and striated muscles. Presence of increased protein in diseased sera may correlate with myocarditis and polymyositis in pSLE patients. This assumption also requires further investigation [27-29].

Transthyretin (TTR) is a serum and cerebrospinal fluid carrier of the thyroid hormone thyroxine (T4) and retinol [30]. TTR variant Del 122 Val protein had a higher expression in pSLE sera compared to healthy controls. Plasma TTR originates primarily from the liver [31]. TTR is known to be associated with the amyloid diseases, senile systemic amyloidosis, familial amyloid polyneuropathy, and familial amyloid cardiomyopathy [32-34]. The presence of this protein in our study suggests that pSLE patients have potential to develop secondary amyloidosis. Sequential quantitation of this protein in long surviving SLE patients and its relationship with development of amyloidosis should be addressed in future studies.

Apolipoprotein A1 (APO A1) is a major constituent of the high density lipoprotein (HDL) complex which acts in cholesterol homeostasis and also has anti-inflammatory properties both in acute and chronic inflammation [35]. Serum apolipoprotein (apo) A-I is also considered to be an immune regulator and can suppress pro-inflammatory cytokines generated by activated T cell in some autoimmune diseases [36]. The present study showed lower expression of apolipoprotein A1 in lupus patients as compared to healthy controls. Zhang, B.*,* et al. (2010) showed lower level of Apo-A-I are seen in SLE and rheumatoid arthritis patients [37]. Further, it has been shown that low levels of this protein are associated with increased risk for cerebrovascular disease and atherosclerosis [38]. This interesting finding in our cohort re-iterates that in future therapeutic modulation of this protein may prevent long term complications of SLE due to exaggerated atherosclerosis.

## Conclusions

In this preliminary proteomic study performed on pooled sera of pSLE patients with active disease, we identified some significant differentially expressed proteins relevant to a pSLE cohort. All novel proteins need validation by ELISA, western blot or by high throughput protein microarray technology**.** Furthermore, the role of these proteins as prognostic markers in adult and pediatric SLE patients needs further investigation.

## Abbreviations

pSLE: Paediatric Systemic Lupus Erythematosus; 2DPAGE: 2 Dimensional Poly Acryl amide Gel Electrophoresis; MALDI-TOF: Matrix Assisted Laser Desorption Ionization Time of Flight; LCMS: Liquid Chromatography Mass Spectrometry; RBP: Retinol Binding Protein; LMOD2: Leiomodin 2; TTR: Transthyretin; Apolipoprotein A1: APOA1.

## Competing interests

The authors declare that they have no competing interests.

## Authors’ contributions

AR executed the study, compiled and analyzed the data, and wrote the manuscript. RWM designed the study, supervised the study, helped with data analysis and edited the manuscript. RA edited the paper, supervised the work and compilation of data. SS supervised part of proteomic work in her lab. SA helped in execution of work. NP helped in execution of the work and compilation of data. SuS participated in the design of the study and helped to draft the manuscript and provided all samples and clinical demographic and laboratory data of pediatric lupus patients. All authors read and approved the final manuscript.
